# Identification of Emerging Organic Pollutants in Aquatic Environments Under the Omics-Based Framework: A Review

**DOI:** 10.3390/molecules31091495

**Published:** 2026-04-30

**Authors:** Xiaotian Zhang, Biao Wang, Xingyue Tu, Qin Zhang, Dan Song, Shasha Liu

**Affiliations:** 1 School of Energy and Environmental Engineering, University of Science and Technology Beijing, Beijing 100083, China; 2 Chinese Research Academy of Environmental Sciences, Beijing 100012, Chinatu.xingyue@craes.org.cn (X.T.); 3 Chongqing Ecological and Environmental Monitoring Center, Chongqing 401336, China; 4 Chongqing Academy of Ecology and Environmental Sciences, Chongqing 401147, China

**Keywords:** emerging organic pollutants, high-resolution mass spectrometry, non-target screening, suspect screening, effect-directed analysis, environmental omics, machine learning

## Abstract

Emerging organic pollutants (EOPs) in aquatic environments have attracted increasing attention because many occur at trace levels, undergo transformation during environmental transport, and contribute to poorly resolved mixture risks. Traditional targeted analysis is inherently restricted to predefined compounds, whereas high-resolution mass spectrometry (HRMS)-based full-scan workflows provide broader opportunities for discovering known unknowns and previously unrecognized contaminants. This review critically synthesizes an omics-based analytical framework for aquatic environments, covering sample digitalization, instrumental analysis and acquisition modes, chemical fingerprint/non-target screening, suspect screening, effect-directed analysis, and confidence-based structural identification. Particular emphasis is placed on practical decision points and trade-offs, including dissolved versus particulate-associated analytes, LC-HRMS versus GC-HRMS coverage, hard versus soft ionization, DDA- versus DIA-type acquisition, database dependence, and the persistent difficulty of linking analytical features to toxicological relevance. The review also discusses emerging directions involving artificial intelligence, chemometrics, organometallic contaminants, and microplastic-associated chemicals. By clarifying conceptual boundaries and highlighting current limitations, this article aims to support the development of more critical, transparent, and risk-oriented workflows for the discovery and prioritization of emerging pollutants in aquatic environments.

## 1. Introduction

The safety of aquatic environments is fundamental to maintaining ecosystem stability and protecting human health. Aquatic systems receive contaminants from industrial effluents, domestic sewage, agricultural runoff, atmospheric deposition, and diffuse urban sources, while environmental transformation processes can generate secondary transformation products with structures and toxicities that remain poorly characterized [1,2,3,4,5,6,7,8]. As a result, aquatic pollution is no longer dominated only by a limited set of legacy compounds, but by dynamic mixtures containing parent chemicals, metabolites, byproducts, and matrix-associated contaminants.

The precise control and risk prevention of emerging pollutants therefore rely on analytical approaches that can move beyond routine monitoring of a predefined list of regulated substances. Current workflows generally involve three complementary analytical logics: targeted analysis for confirmed compounds, suspect screening for expected but not fully confirmed compounds, and non-target screening (NTS) for unexpected or previously unrecognized signals [2,5,9,10,11,12,13]. These approaches are increasingly supported by HRMS, as full-scan acquisition preserves broader chemical information than conventional targeted methods.

Early environmental applications of mass spectrometry relied largely on low-resolution instruments and library-oriented workflows, which remain valuable for the targeted quantification of known compounds. However, their main limitations lie less in mass accuracy alone than in the restricted information available for unknown-feature prioritization, formula assignment, and mixture interpretation. In particular, discrimination among isomeric compounds depends strongly on chromatographic separation and, for some classes, orthogonal information such as ion mobility or authentic standards, rather than on mass resolving power alone [14,15,16].

Recent reviews have covered NTS in water analysis, data processing, and environmental exposure science [10,17,18]. However, the literature still tends to treat sampling, instrumental acquisition, feature prioritization, and risk interpretation as relatively separate components. In addition, some reviews focus primarily on analytical capability, whereas the practical question faced by environmental scientists is often strategic: which workflow is most appropriate for a given decision context, what uncertainties does it resolve, and what new uncertainties does it introduce?

This review defines the “omics-based framework” as an HRMS-centered, full-scan, feature-rich analytical paradigm that extends beyond conventional non-target workflows in two ways. First, it explicitly integrates digitalization, prioritization, structural annotation, and interpretation across the whole workflow rather than treating feature detection as the endpoint. Second, it links analytical outputs to risk-oriented decision support by combining chemistry-driven, list-driven, and effect-driven prioritization strategies. Accordingly, the objectives of this review are to: (1) critically examine analytical workflows from sampling and sample preparation to chromatographic separation, ionization/acquisition modes, and feature extraction; (2) clarify the conceptual boundaries and practical complementarity of chemical fingerprint/non-target screening, suspect screening, and effect-directed analysis; (3) summarize current practices for confidence-based structural annotation, including the distinction between Schymanski Levels 2a and 2b; and (4) synthesize major practical bottlenecks, including matrix effects, inter-platform variability, incomplete databases, and the difficulty of translating detection into risk-relevant interpretation. By emphasizing strengths, limitations, and decision contexts, this review aims to offer a more critical and application-oriented framework for aquatic contaminant discovery and prioritization.

## 2. Omics-Based Analytical Framework for Emerging Pollutants in Aquatic Environments

**Figure 1:**
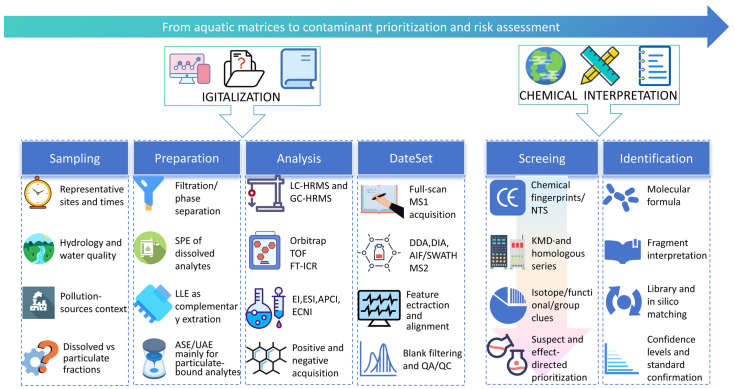
Environmental omics analytical framework.

In this review, the omics-based framework refers to an HRMS-centered workflow that converts complex aquatic samples into feature-rich digital chemical datasets and then interprets those datasets through prioritization, annotation, and risk-relevant reasoning. Figure 1 summarizes the overall workflow, covering representative sampling and preparation, data generation, screening, and structural interpretation [19,20].

### 2.1. Digital Characterization of Aquatic Environmental Samples Using HRMS

#### 2.1.1. Sample Collection and Pre-Treatment Techniques

The value of HRMS-based workflows is determined not only by instrument performance but also by whether the sampling design adequately captures spatial, temporal, and matrix heterogeneity. Representative sampling should therefore consider hydrology, water chemistry, pollution-source context, and the partitioning of contaminants between dissolved and particulate phases. This distinction is especially important because dissolved contaminants and particulate-associated contaminants often require different enrichment and clean-up strategies [5,21,22].

For the dissolved fraction, solid-phase extraction (SPE) remains the most established enrichment strategy because it offers broad applicability, relatively low solvent consumption, and compatibility with downstream LC-HRMS workflows [13,23,24]. Nonetheless, SPE is not universally applicable, as sorbent chemistry, pH, sample volume, salinity, and matrix co-extractives all influence analyte recovery and selectivity. Therefore, an “omics-like” extraction strategy is better understood as a complementary SPE design using multiple sorbents or eluents to expand polarity coverage rather than as a single exhaustive method [14,25,26]. Liquid–liquid extraction (LLE) can provide complementary coverage for selected compound classes, but its solvent demand, emulsion formation, and lower suitability for highly polar analytes limit routine large-scale application in water screening [25,26].

By contrast, accelerated solvent extraction (ASE/PLE) and ultrasonic-assisted extraction (UAE) are more appropriately discussed in relation to particulate matter retained on filters, suspended solids, sediments, or other solid-associated fractions from aquatic samples. These techniques can efficiently recover contaminants sorbed to particulate matrices, but they cannot replace enrichment strategies designed for truly dissolved analytes in bulk water [14,27,28]. In practice, the analytical challenge is therefore not to identify one universally optimal extraction method, but to align extraction selectivity with the environmental question, the matrix fraction, and the intended downstream screening strategy.

Minimalist clean-up is often desirable in NTS-oriented studies because aggressive purification can remove low-abundance or structurally unusual compounds together with interferences. At the same time, insufficient clean-up can exacerbate ion suppression, background contamination, and false positives. The choice must therefore be balanced rather than categorical, especially for multi-class screening in wastewater, drinking water, and complex coastal matrices [2,13,23,24].

#### 2.1.2. Instrument Analysis, Data Acquisition, and Data Mining

Instrument selection should be driven by chemical space, not by the assumption that any single HRMS platform is universally comprehensive. LC-HRMS is generally preferred for polar, ionic, thermolabile, and non-volatile contaminants, whereas GC-HRMS is advantageous for volatile, semi-volatile, and less polar compounds after suitable sample preparation and, when necessary, derivatization [29,30]. These platforms should therefore be regarded as complementary rather than interchangeable. In aquatic studies, combining LC-HRMS and GC-HRMS can substantially enlarge analyte coverage, but at the cost of higher instrument demand, greater data complexity, and more difficult cross-platform harmonization [5,30,31].

Ionization mode further governs which compounds are visible and how interpretable their spectra are. In GC-MS, hard ionization such as electron ionization (EI) produces reproducible fragmentation and strong library compatibility, which is advantageous for annotation and retrospective mining. However, extensive fragmentation may reduce molecular-ion abundance for some compounds. In contrast, softer ionization strategies such as APCI or other atmospheric-pressure interfaces can preserve molecular ions and improve formula assignment for selected contaminant classes, but may provide less standardized fragmentation behavior [32,33]. In LC-MS, electrospray ionization (ESI) offers broad sensitivity for polar compounds but is strongly affected by matrix effects and adduct formation, whereas APCI can be beneficial for less polar analytes and sometimes produces simpler ion patterns [33,34,35]. The practical implication is that data-mining strategies differ between ionization regimes: EI-based workflows often rely heavily on deconvolution and spectral-library matching, while ESI-based workflows require careful treatment of adducts, in-source fragments, isotope clusters, and blank subtraction [36,37].

Data acquisition mode has a direct impact on the amount, quality, and interpretability of MS/MS evidence. Full-scan MS1 data are essential for feature discovery and retrospective analysis, but structural interpretation depends heavily on how MS2 information is generated [36]. Data-dependent acquisition (DDA; e.g., auto-MS/MS) tends to provide cleaner compound-specific product-ion spectra and is often advantageous for suspect screening when a manageable number of prioritized features can trigger MS/MS events. Its main limitation is stochastic precursor selection, which can bias coverage toward high-intensity features and reduce reproducibility across runs. By contrast, data-independent acquisition (DIA), including all-ions fragmentation (AIF) and SWATH-type approaches, can improve MS2 coverage for complex samples and better support broad non-target screening, but the resulting fragment spectra are more difficult to deconvolute because fragments from multiple coeluting precursors may be mixed [36,38].

Accordingly, no single acquisition mode is optimal for all screening purposes. For knowledge-guided suspect screening, DDA often offers higher spectral specificity and easier downstream confirmation. For discovery-oriented NTS, DIA-type acquisition may improve coverage, but only if data processing is sufficiently robust to disentangle fragment relationships. In practice, hybrid strategies are increasingly attractive: broad MS1 acquisition is followed by iterative DDA reinjection, inclusion-list MS/MS, or targeted confirmation for prioritized features. This staged design reduces the false sense of completeness that can arise from any single acquisition mode [36,39].

Beyond spectral acquisition, modern omics workflows depend on advanced data mining. Common steps include peak detection, alignment, blank filtering, isotope/adduct grouping, statistical prioritization, and library or in silico annotation [37]. Chemometric tools such as principal component analysis, partial least squares-discriminant analysis, hierarchical clustering, and multiblock multivariate integration can reveal latent structure in complex feature matrices, while machine-learning approaches increasingly support feature prioritization, source identification, and pattern recognition [40,41,42,43]. However, these tools do not eliminate analytical uncertainty; instead, they relocate the challenge toward model validation, interpretability, and overfitting control.

### 2.2. Priority Screening Strategies for Emerging Pollutants in Aquatic Environments

**Figure 2:**
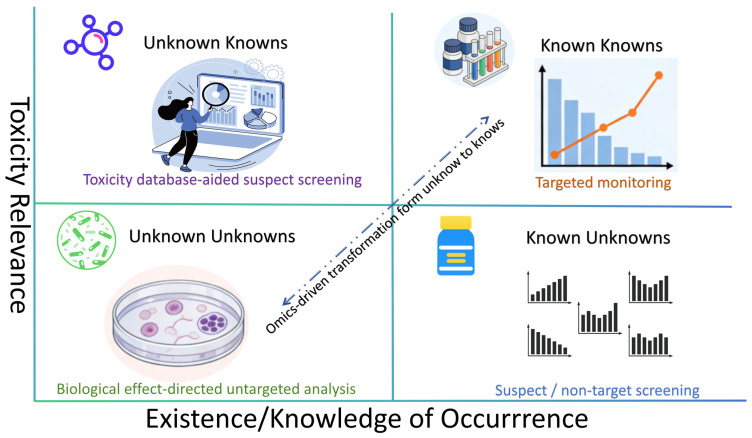
Integrated screening strategies in full-component data space.

A key conceptual clarification is that suspect screening should not simply be treated as a subset of non-target screening. Instead, the three major prioritization logics discussed here are better viewed as parallel but interacting strategies: (i) chemical fingerprint or feature-driven NTS, which starts from detected unknown features; (ii) suspect screening, which starts from a predefined candidate list; and (iii) effect-directed analysis (EDA), which starts from observed biological activity. These strategies address different analytical questions, resolve different uncertainties, and introduce different biases. Figure 2 and Table 1 summarize their relationships.

#### 2.2.1. Chemical Fingerprint Screening and Feature-Driven NTS

Chemical fingerprinting refers to the use of feature patterns such as accurate mass, isotope signatures, mass defects, homologous-series relationships, characteristic neutral losses, and diagnostic fragments to prioritize unknowns. It overlaps with NTS because both begin with observed features rather than predefined compound lists, but the term “chemical fingerprinting” is useful when the emphasis is on chemistry-guided prioritization rather than exhaustive feature enumeration [39,44,45].

Tools such as Kendrick mass defect (KMD) analysis are particularly effective for classes that exhibit repeating structural units, for example, PFAS-related series, surfactants, or some additive-derived transformation products [46,47,48]. Nevertheless, KMD is not universally informative: its performance depends on signal quality, matrix complexity, and whether the compound class actually conforms to homologous behavior. Similarly, isotope profiling can be powerful for halogenated contaminants and selected organometallic species, but interpretation becomes less straightforward when multiple adducts, coeluting isobars, or low-abundance isotopologues are involved [44,45,48,49,50,51,52].

The organometallic field illustrates both the potential and the limits of fingerprint-based approaches. Organoiodine and organosilicon pollutants can sometimes be prioritized using exact-mass and isotope-pattern logic, but structure elucidation often requires complementary evidence from chromatographic behavior, fragmentation, and, in some cases, element-specific methods such as HPLC-ICP-MS or orthogonal spectroscopic tools [19,20,49,53,54]. Organotin compounds further demonstrate that occurrence alone is not enough: speciation and toxicity information are crucial because different organometallic forms can differ substantially in persistence, bioavailability, and hazard [53].

Microplastic-associated contaminants provide another important frontier. In aquatic systems, the analytical target may include polymer particles, intentionally added additives, non-intentionally added substances (NIAS), sorbed contaminants, and transformation products released during weathering. Omics-oriented workflows are particularly useful for the dissolved additive and leachate fraction, where suspect and non-target screening can capture a broader chemical profile than polymer-identification tools alone [55,56]. However, the particle itself may require other techniques such as Py-GC-MS, FTIR, Raman, or microscopy-based workflows, reminding us that no single ‘omics’ method can fully represent the entire chemical and particulate burden of microplastics.

#### 2.2.2. Suspect Screening

Suspect screening is a knowledge-guided strategy in which HRMS data are interrogated against a predefined candidate list, typically using exact mass, isotope pattern, retention behavior, and fragment evidence [2,57,58]. Because the workflow begins with candidate identities, suspect screening is usually more efficient and interpretable than open-ended NTS, especially for compounds with regulatory relevance or plausible occurrence in a given catchment. This is one reason why suspect screening has become central in aquatic monitoring for PFAS, flame retardants, pharmaceuticals, pesticides, additives, and disinfection byproducts [27,48,59,60,61,62].

Its key weakness, however, is the presence of structural blind spots. The method can only find what is represented in the suspect list or what is structurally close enough to be inferred by the analyst. Incomplete databases, inconsistent identifiers, missing transformation products, and poor metadata quality can therefore generate false negatives even when the instrument has detected a relevant signal [63,64,65]. This is especially important for rapidly evolving contaminant spaces such as microplastic-derived additives, NIAS, and mixture-related transformation products, where list completeness lags behind real-world emissions [56].

For this reason, suspect screening should be viewed as a prioritization accelerator rather than a replacement for broader discovery. In practical workflows, suspect screening is most powerful when embedded within a staged design: list-based annotation is applied first, then unresolved or biologically relevant features are passed forward to NTS or EDA. This combined strategy reduces interpretive burden while preserving the possibility of discovering unexpected contaminants.

#### 2.2.3. Biological Effect-Directed Screening

Effect-directed analysis (EDA) shifts the starting point from chemical occurrence to observed bioactivity. In this approach, extracts or fractions are evaluated with bioassays, and the chemically most relevant features are prioritized because they co-occur with biological effects such as endocrine disruption, oxidative stress, mutagenicity, developmental toxicity, or enzyme inhibition [3,8,66,67,68,69,70,71,72,73,74,75,76,77]. This makes EDA especially attractive for risk prioritization, since the most abundant compound is not necessarily the one that contributes most to biological effects.

At the same time, EDA is analytically demanding. Fractionation increases sample-handling complexity, may dilute weakly active compounds, and can change mixture composition. Bioassay throughput, endpoint specificity, and compatibility with fraction volumes also constrain study design. Most importantly, linking an observed effect to a unique causative compound is often difficult because multiple chemicals may contribute additively or interactively to the same endpoint [3,66,78].

Thus, EDA should not be framed as a universal solution but as a strategically powerful complement. It is most valuable when the central question concerns hazard-relevant prioritization, for example, in wastewater effluents, drinking-water treatment trains, microplastic leachates, or complex environmental mixtures where occurrence-based ranking alone is insufficient.

### 2.3. Chemical Structure Identification of Emerging Pollutants in Aquatic Environments

Confidence communication remains essential because most HRMS-based studies do not deliver fully confirmed structures for every prioritized feature. The Schymanski framework therefore remains the most widely used system for reporting annotation confidence in environmental HRMS [11,79].

Level 5 indicates an exact mass of interest only. Level 4 indicates an unequivocal molecular formula. Level 3 corresponds to tentative candidate structure(s). Importantly, Level 2 should be subdivided into Level 2a (probable structure via library spectrum match) and Level 2b (probable structure via diagnostic evidence such as fragments, knowledge of the literature, or class reasoning without a direct library spectrum match). Level 1 requires confirmation with an authentic standard, including retention time and spectral agreement [79].

This distinction between Levels 2a and 2b is more than a minor technical detail. In practice, it separates candidates supported by strong reference-spectrum evidence from those supported by plausible but less definitive reasoning. Because environmental studies often rely on incomplete libraries and unavailable standards, many annotations remain at Levels 2–4. This should be explicitly acknowledged as a central limitation of current workflows rather than as a routine interim step that can be assumed away [79,80,81,82,83].

Future progress in structure elucidation will depend on better spectral libraries, retention-index and collision-cross-section databases, in silico fragmentation tools, and orthogonal confirmation strategies combining HRMS with approaches such as ion mobility, NMR, or element-specific detection [37,53,81,83,84].

### 2.4. Current Limitations and Practical Challenges

Several limitations recur across the entire omics workflow. First, matrix effects remain pervasive. Extraction recovery, ion suppression, adduct formation, and background contamination all reshape feature intensity and can distort comparisons among sites, times, or laboratories. This means that apparent differences in feature abundance are not always environmentally meaningful [17,37,85].

Second, analytical breadth and interpretability are in tension. Expanding chemical coverage through multi-sorbent extraction, multi-platform HRMS, or DIA-type acquisition may increase discovery potential, but it also expands the burden of deconvolution, curation, and false-positive control. An increase in data volume does not automatically translate into more reliable knowledge.

Third, prioritization strategies are not interchangeable. Feature-driven NTS broadens discovery but inflates annotation burden; suspect screening improves efficiency but inherits database bias; EDA improves toxicological relevance but introduces fractionation and causality challenges. Strong workflows therefore depend on transparent justification for why a particular strategy was chosen for a specific decision context.

Fourth, AI and chemometrics are increasingly influential but should be applied cautiously. Machine-learning models can support feature ranking, source apportionment, and mixture interpretation, yet their outputs remain highly sensitive to preprocessing choices, training data quality, class imbalance, and external validation [40,41,42,43]. Emerging AI paradigms, such as federated learning and neuro-symbolic learning, may offer useful inspiration for distributed data integration and more interpretable model reasoning, but their transfer to environmental omics still requires domain-specific validation [86].

Finally, translating HRMS-based detection into actionable risk assessment remains challenging. Detection alone does not establish environmental significance, because many prioritized features still lack robust information on sources, persistence, exposure pathways, bioaccumulation, and mixture effects. This limitation is especially evident for organometallic species, transformation products, and microplastic-associated chemicals, for which occurrence data often outpace toxicological and fate-related evidence. In practice, risk interpretation therefore requires a staged approach that links analytical prioritization with targeted confirmation, exposure characterization, and effect-relevant follow-up, rather than assuming that feature detection can be directly converted into management decisions [7,54,55,56].

## 3. Conclusions and Perspectives

The omics approach centered on HRMS has substantially expanded the analytical horizon for emerging pollutants in aquatic environments. Through optimization across the full workflow—from sample collection and pretreatment to instrumental analysis, data acquisition, prioritization, and structural annotation—it enables the comprehensive digital characterization of complex aquatic environmental matrices and provides an important analytical basis for the discovery of previously unrecognized contaminants [10,16,85]. More importantly, its value lies not simply in generating more analytical features, but in combining broad chemical coverage with transparent prioritization logic and confidence-based interpretation.

Within this framework, chemical fingerprint/feature-driven non-target screening, suspect screening, and effect-directed analysis should be regarded as complementary but distinct prioritization strategies rather than interchangeable labels. Chemical fingerprinting and feature-driven NTS offer broad discovery potential for unexpected compounds and transformation products; suspect screening improves efficiency and interpretability for expected or knowledge-guided contaminants; and EDA introduces toxicological relevance into the prioritization process, which is especially important for complex mixtures in aquatic environments [3,37,74,75]. At the same time, confidence-based structural identification remains essential. The Schymanski framework, including the distinction between Levels 2a and 2b, provides an important basis for transparent communication of annotation certainty, particularly because many environmental HRMS studies still cannot routinely achieve full Level 1 confirmation [78,79,80,81,82,83].

To further advance the application of omics methods in studies of emerging pollutants in aquatic environments, future work should focus on a limited set of priorities that address current analytical bottlenecks and, in particular, the gap between contaminant detection and decision-relevant interpretation [87,88].

**Figure 3:**
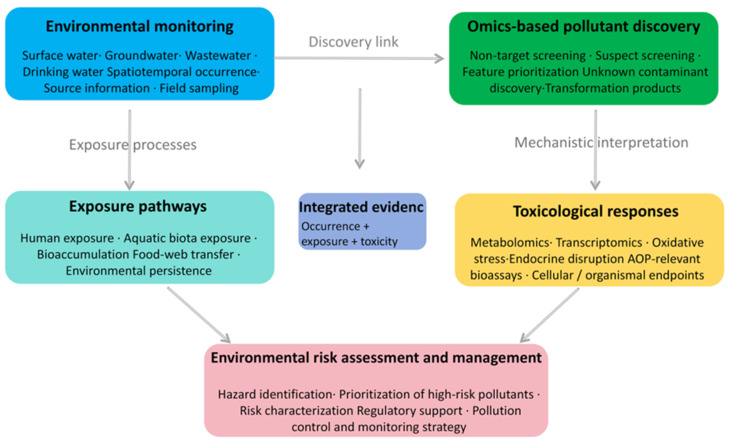
Pollutant discovery–risk conceptual framework.

1. Synergistic Development of Instrumental Technologies: Promote the deeper integration of chromatographic separation and HRMS to improve analytical coverage, selectivity, and confidence in structure elucidation. The development of fully two-dimensional chromatography systems, such as GC × GC-HRMS and LC × LC-HRMS, can enhance the separation of isomers and complex mixtures. Online analytical technologies may also improve the monitoring of unstable and trace contaminants in aquatic environments. In addition, combining HRMS with orthogonal techniques such as ion mobility, nuclear magnetic resonance, Raman spectroscopy, or element-specific detection would provide more robust multidimensional evidence for pollutant characterization, especially for challenging contaminant classes such as organometallic compounds and transformation products [34,35,51,78,79].
2. Refinement of Data Processing and Database Systems: Develop more robust, transparent, and automated data-processing strategies for feature extraction, deconvolution, adduct and isotope annotation, blank subtraction, and cross-platform comparability, so as to improve the reliability of full-component data interpretation. At the same time, dedicated spectral libraries and pollutant databases for aquatic environments should be continuously strengthened by integrating accurate mass, molecular formula, fragmentation behavior, retention-related information, environmental occurrence, transformation pathways, and toxicological relevance. Better curation and standardization of such databases will be critical for reducing false positives and false negatives in both suspect screening and non-target workflows [10,16,35,36,83].
3. Integrated Application of Multidimensional Screening Strategies: Future studies should place greater emphasis on workflow integration at the prioritization stage rather than simply expanding the number of detected features. In practical terms, chemistry-driven NTS, suspect screening, and EDA should be combined according to study objectives, sample complexity, and available confirmation capacity. In this context, chemometric and artificial-intelligence tools may support feature ranking, source identification, and pattern recognition, but only when preprocessing procedures, training data, and validation strategies are clearly reported. Optimization strategies developed in complex decision-making systems, such as receding horizon optimization and differential evolution algorithms, may also provide methodological inspiration for adaptive feature prioritization and multi-objective decision support in future environmental omics workflows. However, their application should be validated using domain-specific environmental datasets before they are introduced into practical screening or risk-assessment workflows [89]. Greater methodological attention is also needed for under-characterized contaminant spaces, particularly organometallic pollutants and microplastic-associated chemical mixtures, where current omics workflows remain promising but still analytically fragmented [37,38,39,40,41,51,52,53,54,74,75,90].
4. Integration of Identification and Risk Assessment: A major next step is to narrow the gap between contaminant identification and risk interpretation. Rather than treating HRMS outputs as self-sufficient evidence, future studies should connect prioritized contaminants with source information, environmental fate, bioaccumulation potential, mixture toxicity, and exposure-relevant endpoints to support more defensible environmental decision-making [40,53,77,91,92]. As illustrated in Figure 3, the proposed pollutant discovery–risk conceptual framework should be understood as a stepwise structure for linking monitoring data, omics-based discovery, exposure pathways, and toxicological responses, rather than as a universal solution. Its practical value will depend on case-specific implementation, especially through targeted confirmation, effect-based validation, and transparent prioritization criteria.

The management of emerging pollutants in aquatic environments remains a long-term and challenging task. Omics approaches have clearly expanded opportunities for contaminant discovery in complex aquatic matrices, but their contribution to environmental governance will depend less on the expansion of feature numbers than on better comparability, more transparent communication of annotation confidence, and stronger integration with exposure and effect evidence. Continued progress in instrumentation, data processing, database curation, and workflow design is therefore important, but equally important is the development of study designs that convert analytical outputs into defensible prioritization and risk-oriented interpretation [16,85].

## Figures and Tables

**Table 1:** Practical comparison of major prioritization strategies for aquatic emerging pollutants.

Strategy	Main Question	Key Strengths	Main Limitations	Typical Best-Use Scenarios
Chemical fingerprint/NTS	What unexpected features are present?	Broad discovery power; retrospective re-analysis; useful for homologous series and unusual compounds	High annotation burden; feature inflation; matrix and platform dependence	Exploratory surveys, transformation-product discovery, previously unrecognized contaminants
Suspect screening	Are the expected candidate compounds present?	Higher interpretability; easier prioritization; efficient for regulatory and knowledge-guided monitoring	Database incompleteness; false negatives for compounds absent from lists; confirmation still needed	Routine surveillance, targeted expansion lists, source-informed monitoring, known classes such as PFAS or additives
Effect-directed analysis	Which fractions or features are linked to biological activity?	Risk relevance; highlights toxicologically important unknowns; supports prioritization beyond occurrence	Fractionation complexity; assay throughput constraints; causality between effect and compound can remain ambiguous	Mixture toxicity studies, wastewater or leachate prioritization, hazard-oriented discovery

## Data Availability

The data are contained within the article.

## Abbreviations

The following abbreviations are used in this manuscript:

AIF	all-ions fragmentation
AOP	adverse outcome pathway
APCI	atmospheric-pressure chemical ionization
ASE	accelerated solvent extraction
DDA	data-dependent acquisition
DIA	data-independent acquisition
EDA	effect-directed analysis
ECNI	electron capture negative ionization
EI	electron ionization
EOPs	emerging organic pollutants
ESI	electrospray ionization
FT-ICR	Fourier transform ion cyclotron resonance
GC-HRMS	gas chromatography-high-resolution mass spectrometry
HRMS	high-resolution mass spectrometry
KMD	Kendrick mass defect
LC-HRMS	liquid chromatography-high-resolution mass spectrometry
LLE	liquid–liquid extraction
NIAS	non-intentionally added substances
NTS	non-target screening
PFAS	per- and polyfluoroalkyl substances
PLE	pressurized liquid extraction
QA/QC	quality assurance/quality control
SPE	solid-phase extraction
SWATH	sequential window acquisition of all theoretical fragment-ion spectra
UAE	ultrasonic-assisted extraction
